# Supplementary material for: Design, Synthesis and Pharmacological Evaluation of Novel Conformationally Restricted N-arylpiperazine Derivatives Characterized as D2/D3 Receptor Ligands, Candidates for the Treatment of Neurodegenerative Diseases
Source: Biomolecules. 2022 Aug 12;12(8):1112. doi: 10.3390/biom12081112 (PMC9405847; doi:10.3390/biom12081112)
Supplement: Supplementary file 1 [file biomolecules-12-01112-s001.zip › biomolecules-1837816-supplementary.pdf]

Supplementary

1. **Spectral and HPLC data of Synthesized Compounds:**

Figures S1-S4: Spectral and HPLC data of *N*-phenylpiperazine derivative **5a**

Figures S5-S8: Spectral and HPLC data of *N*-phenylpiperazine derivative **5b**

Figures S9-S12: Spectral and HPLC data of *N*-phenylpiperazine derivative **5c**

Figures S13-S16: Spectral and HPLC data of *N*-phenylpiperazine derivative **5d**

Figures S17-S20: Spectral and HPLC data of *N*-phenylpiperazine derivative **5e**

Figures S21-S24: Spectral and HPLC data of *N*-phenylpiperazine derivative **5f**

2. **Molecular Modeling**

Table S1: RMSD values for each of the GOLD program function.

Figure S25: Overlap of risperidone structure of the crystallographic structure (PDB 6CM4) in orange, and the result obtained after redocking by the ChemPLP function in purple.

Table S2: Values of the scores of the results obtained through the docking calculation from the GOLD program, using the ChemPLP function.

Figure S26: Interaction profile of the proposed compounds on D_3 (grey) and D_2 (blue) receptors. A and B: 5a (light blue); C and D: 5b (pink); E and F: 5c (yellow).

Figure S27: Interaction profile of the proposed compounds on D_3 (grey) and D_2 (blue) receptors. A and B: 5d (orange); C and D: 5e (pink); E and F: 5f (purple).

Figure S28: PLIP analysis for the compound 5b, at D_2 receptors.
Figure S29: PLIP analysis for the compound 5b, at D_3 receptors.
Figure S30: PLIP analysis for the compound 5c, at D_2 receptors.
Figure S31: PLIP analysis for the compound 5c, at D_3 receptors.
Figure S32: PLIP analysis for the compound 5d, at D_2 receptors.
Figure S33: PLIP analysis for the compound 5d, at D_3 receptors.
Figure S34: PLIP analysis for the compound 5e, at D_2 receptors.
Figure S35: PLIP analysis for the compound 5e, at D_3 receptors.
Figure S36: PLIP analysis for the compound 5f, at D_2 receptors.
Figure S37: PLIP analysis for the compound 5f, at D_3 receptors.

3. **Binding and GTP-shift**

Figure S38: Estimation of the affinity of of 5a (A), 5b (B), 5c (C), 5d (D), 5e (E) and 5f (F) on [^{3}H]-YM-09151-2 binding to rat striatal D2 receptor (D2R). Data are means (±S.E.) from two or three independent experiments, each performed in triplicate. The data were fitted assuming a single population of binding sites and curves were drawn using the parameters fitted by nonlinear regression (see details in the Materials and Methods).
Figure S39: Estimation of the intrinsic efficacy of 5b (A), 5c (B), 5d (C), 5e (D), and 5f (E) at the human D_3 in membrane preparations of recombinant Chem-1 cells. Competition curves were performed using the antagonist radioligand (0.5 nM [^{3}H]-spiperone) in the presence of 5 mM $MgCl_2$ and 1.5 mM $CaCl_2$ (black) or 154 mM NaCl and 1 mM GTP (blue). Each curve represents the averaged curve (±S.E.) from two or three independent paired experiments (in triplicate).

1. Spectral and HPLC data of Synthesized Compounds:

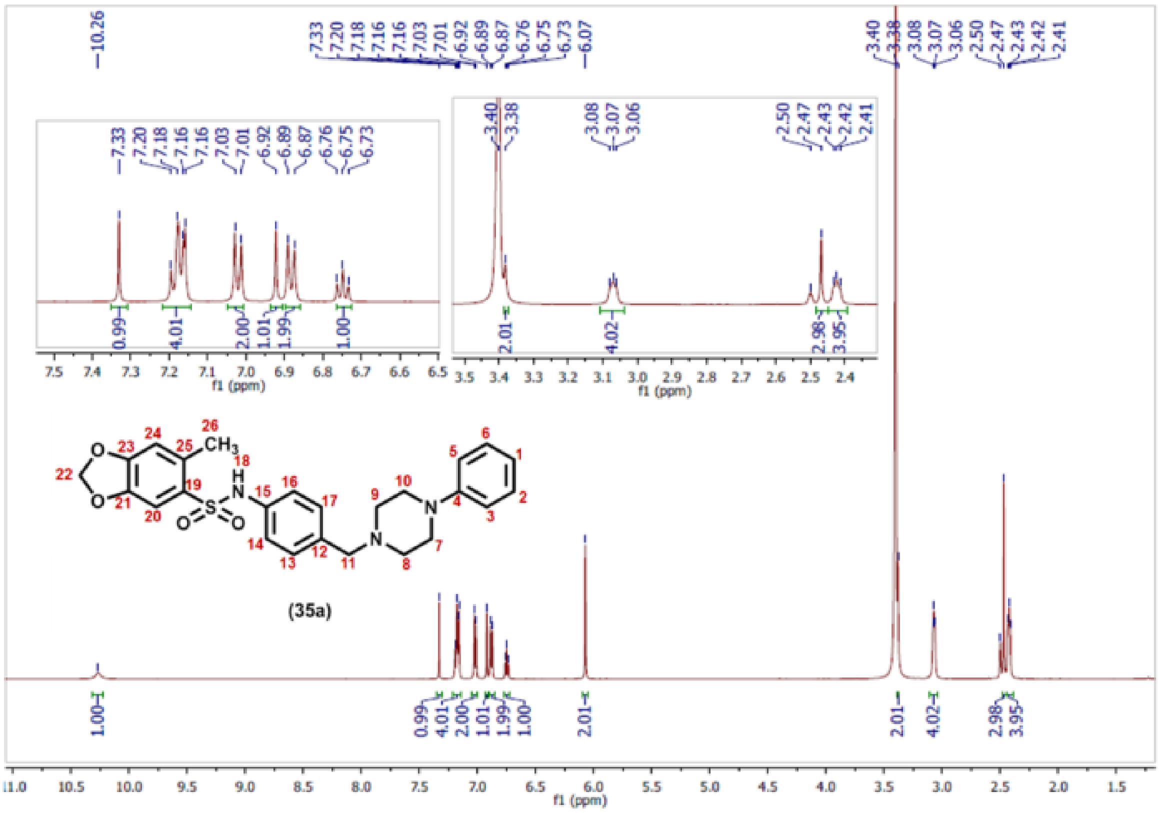

Figure S1: NMR ^{1}H (500 MHz, 25˚C, DMSO-d_6) of **5a**.

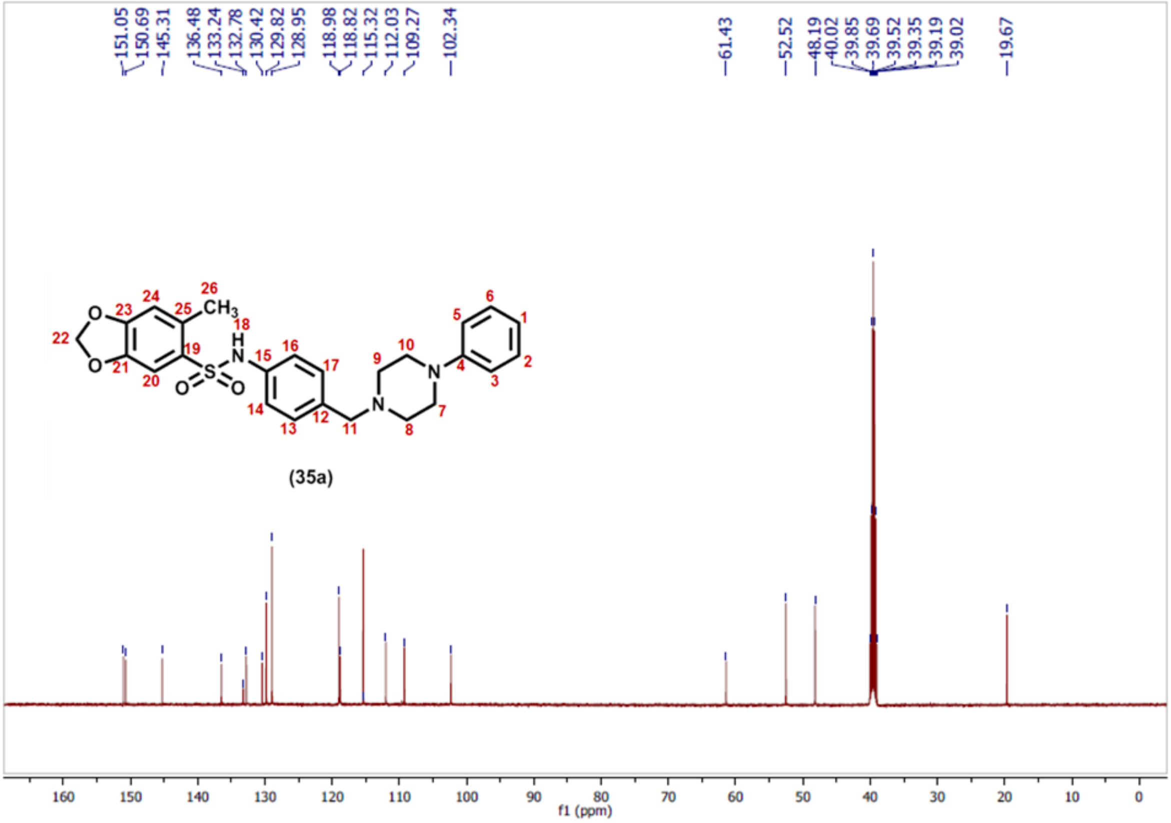

Figure S2: NMR ^{13}C (500 MHz, 25˚C, DMSO-d_6) of **5a**.

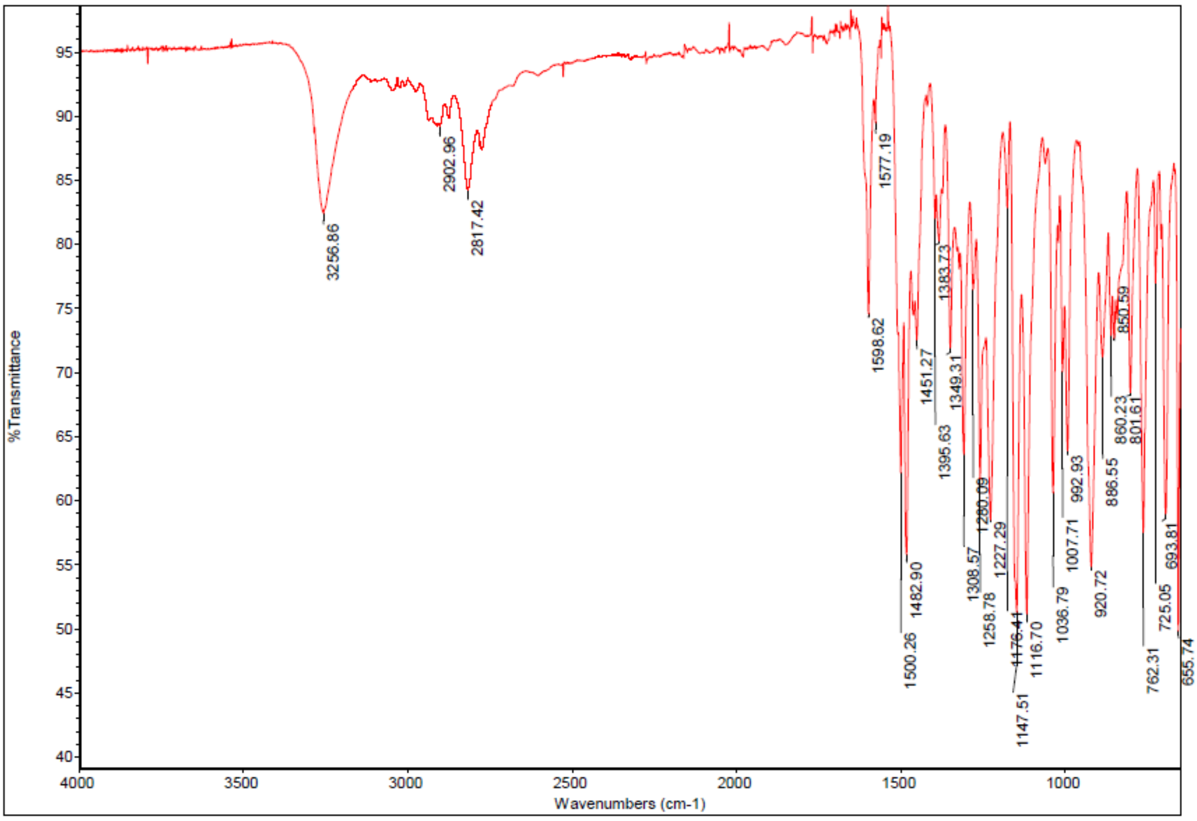

Figure S3: Infrared (ATR) of **5a**.

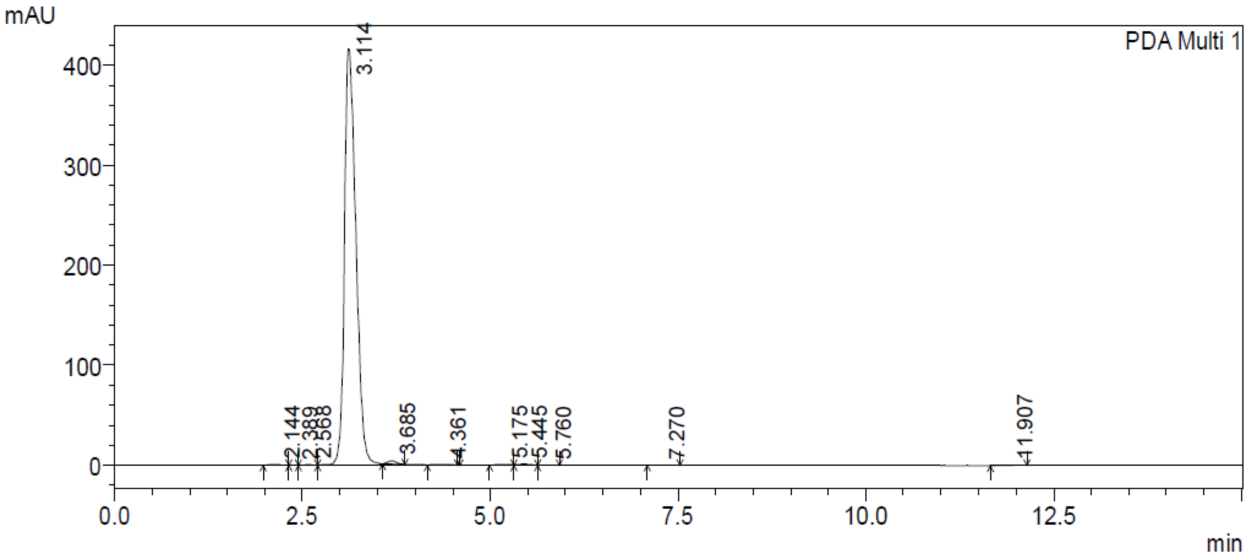

Figure S4: Reverse phase chromatogram in ACN:H2O (60:40) at 254 nm of **5a**.

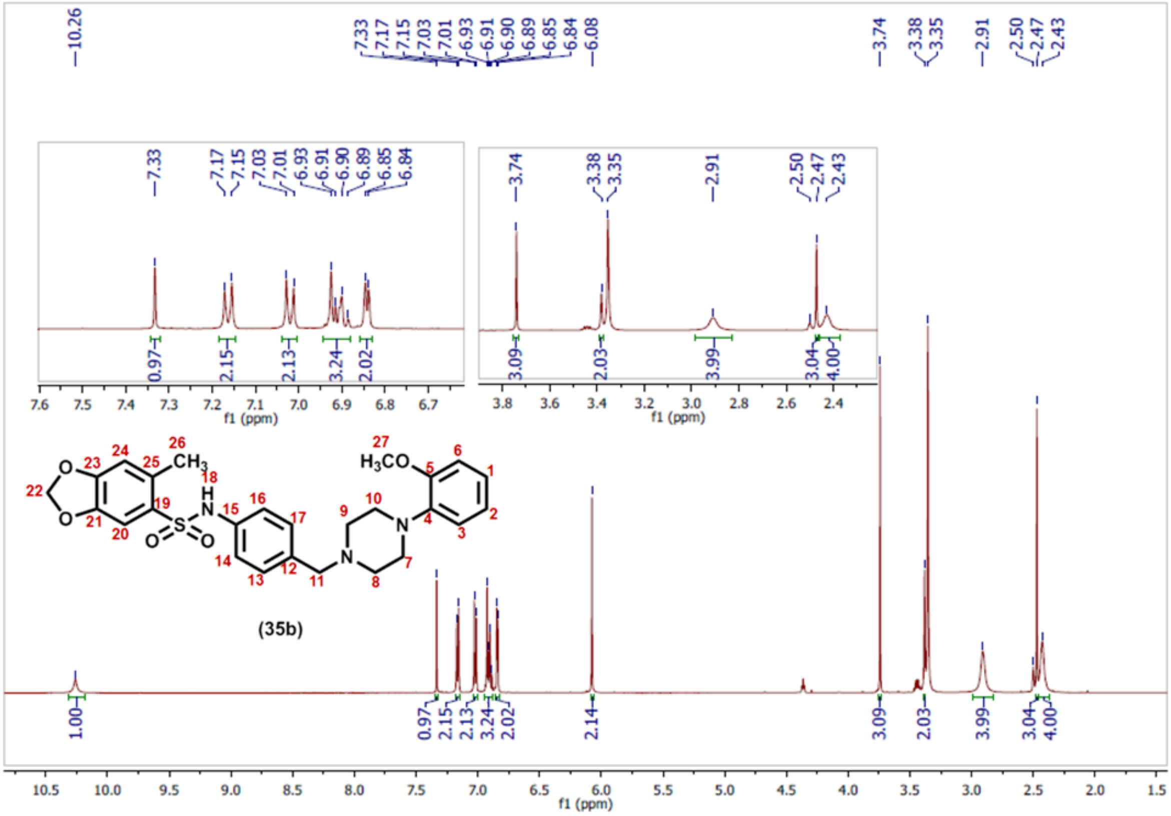

Figure S5: NMR ^{1}H (500 MHz, 25˚C, DMSO-d_6) of **5b**.

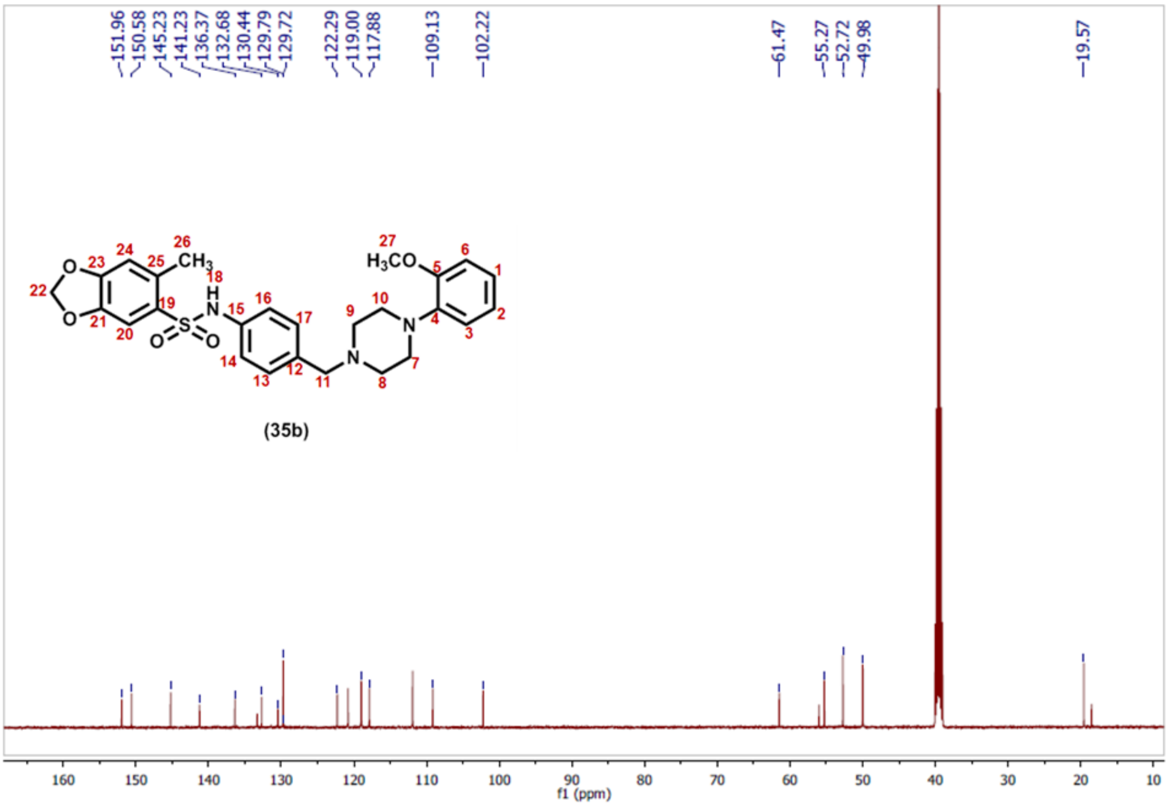

Figure S6: NMR ^{13}C (500 MHz, 25˚C, DMSO-d_6) of **5b**.

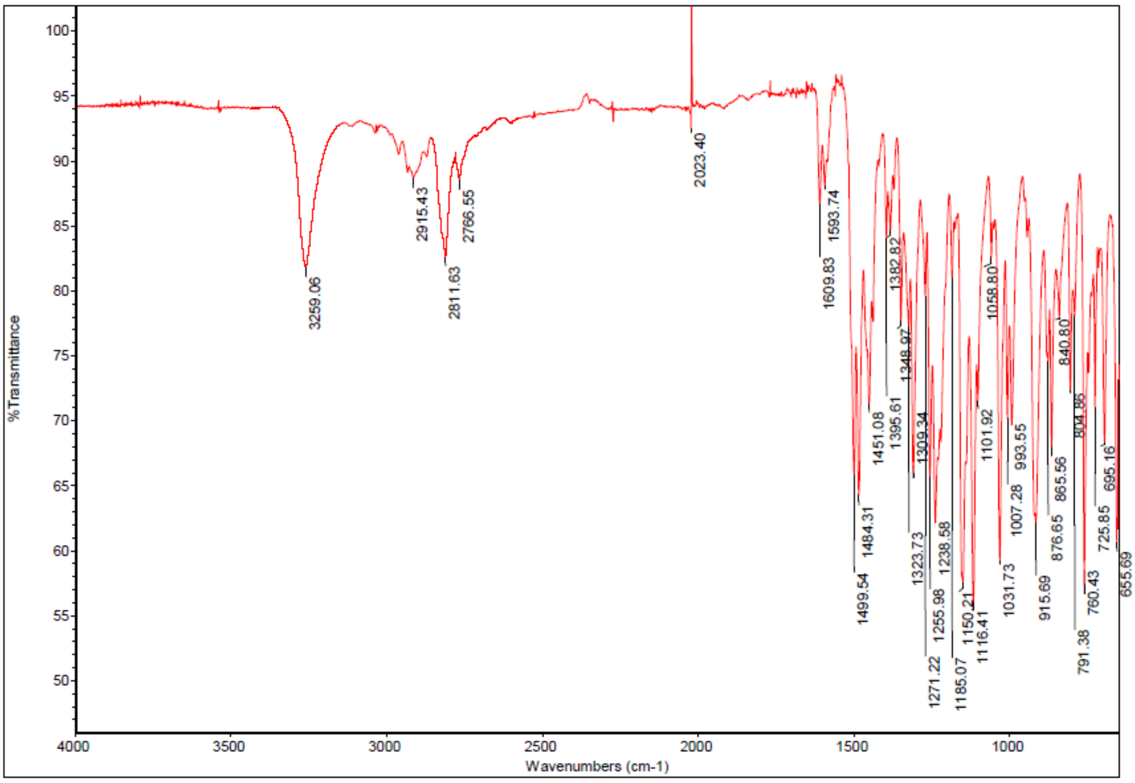

Figure S7: Infrared (ATR) of **5b**.

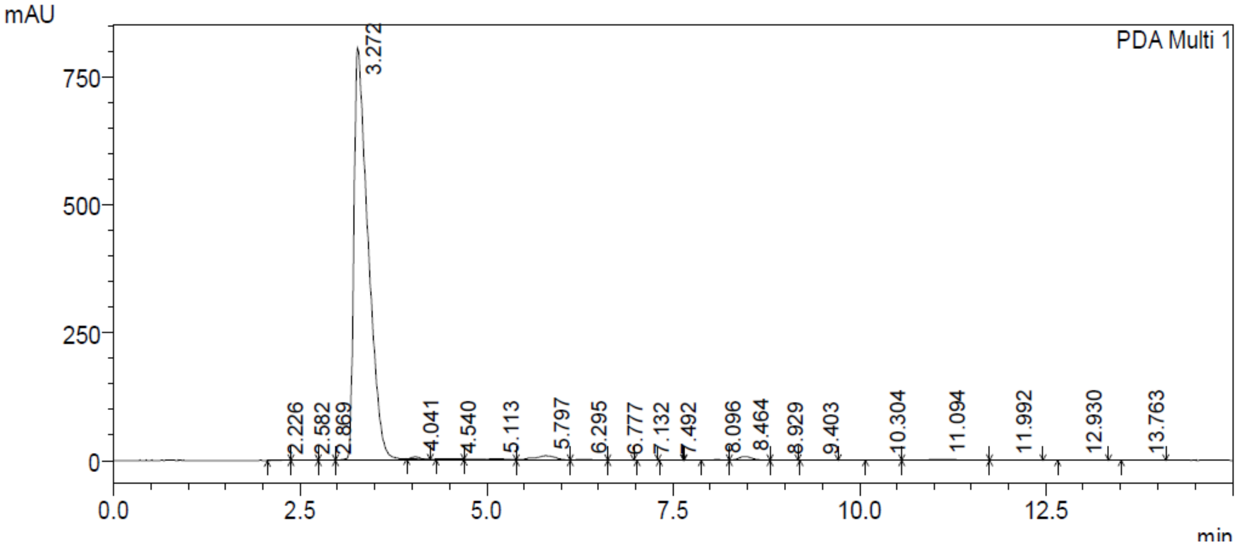

Figure S8: Reverse phase chromatogram in EtOH:H2O (80:20) at 254 nm of **5b**.

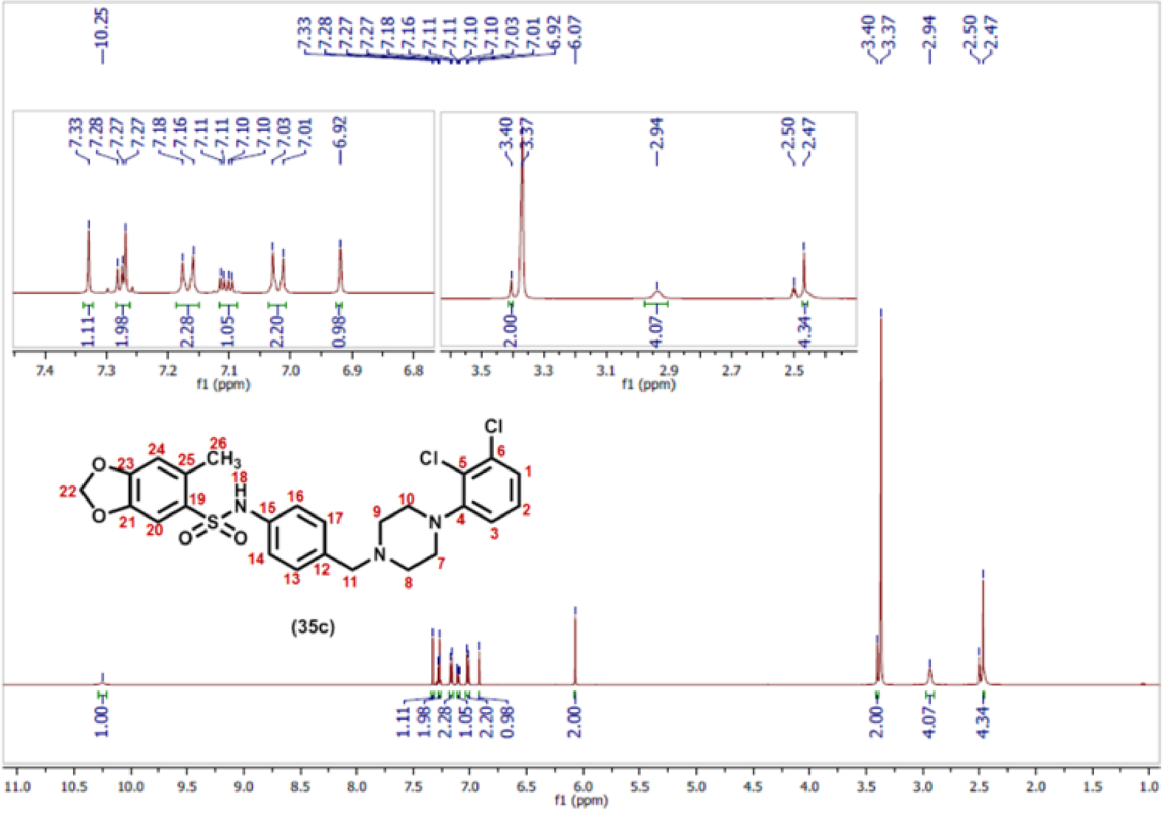

Figure S9: NMR ^{1}H (500 MHz, 25˚C, DMSO-d_6) of **5c**.

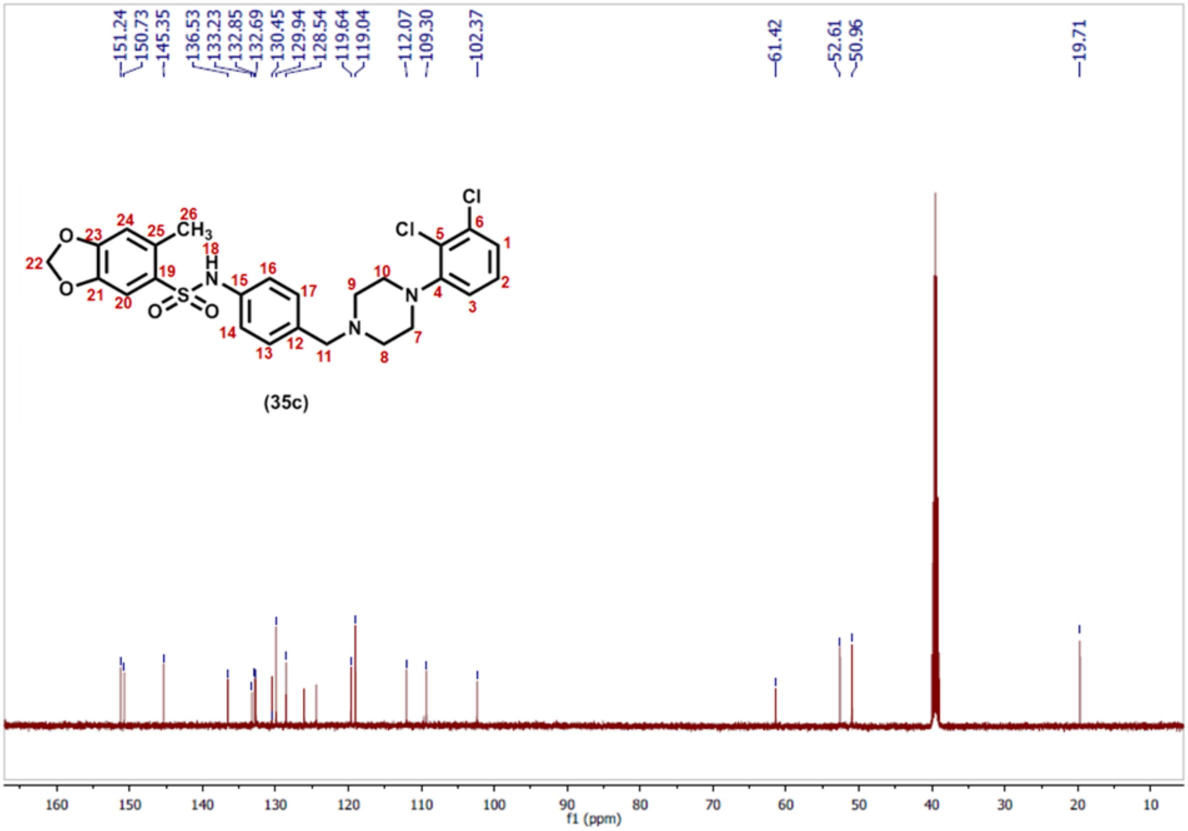

Figure S10: NMR ^{13}C (500 MHz, 25˚C, DMSO-d_6) of **5c**.

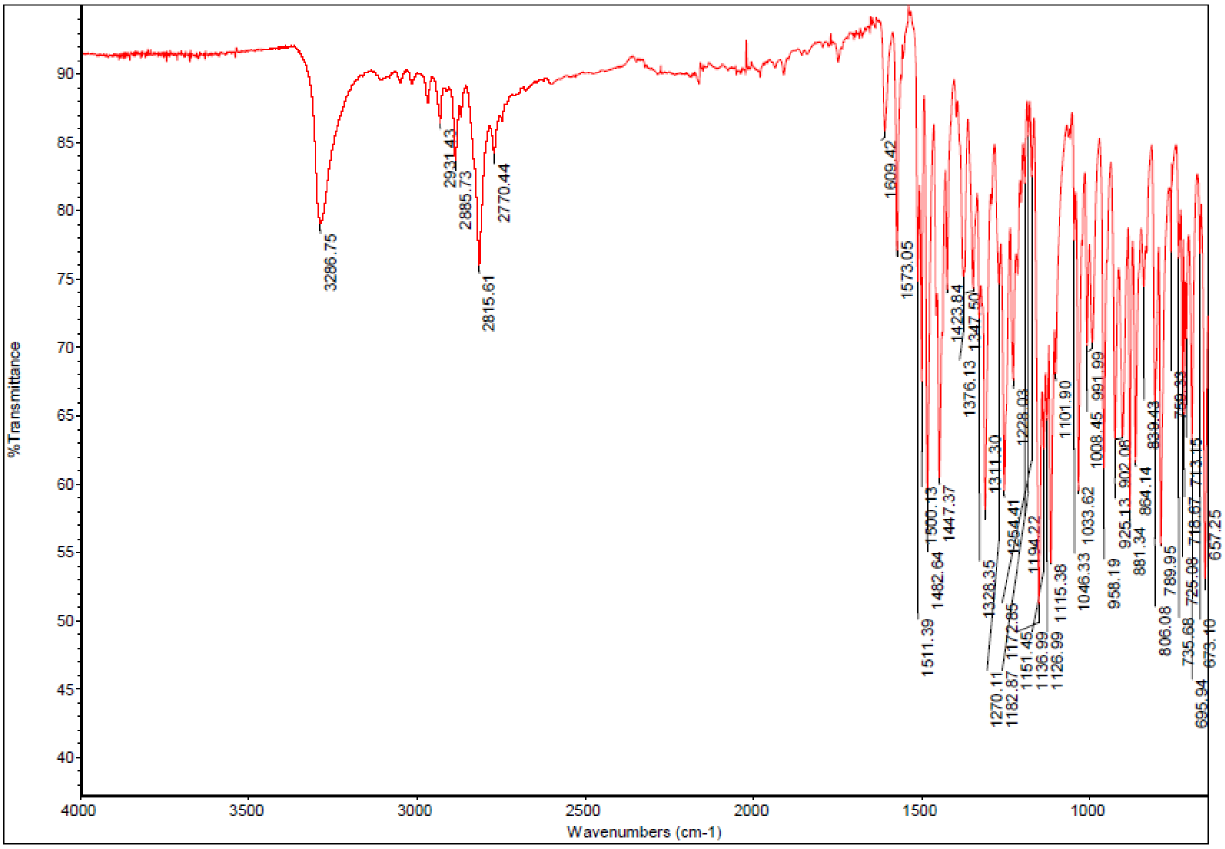

Figure S11: Infrared (ATR) of **5c**.

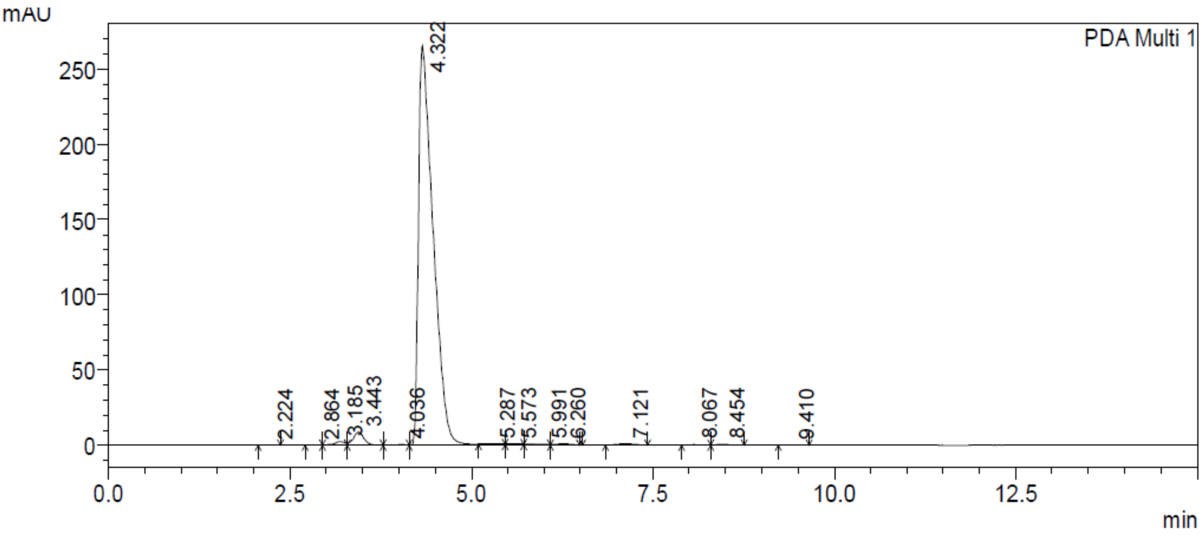

Figure S12: Reverse phase chromatogram in EtOH:H2O (80:20) at 254 nm of **5c**.

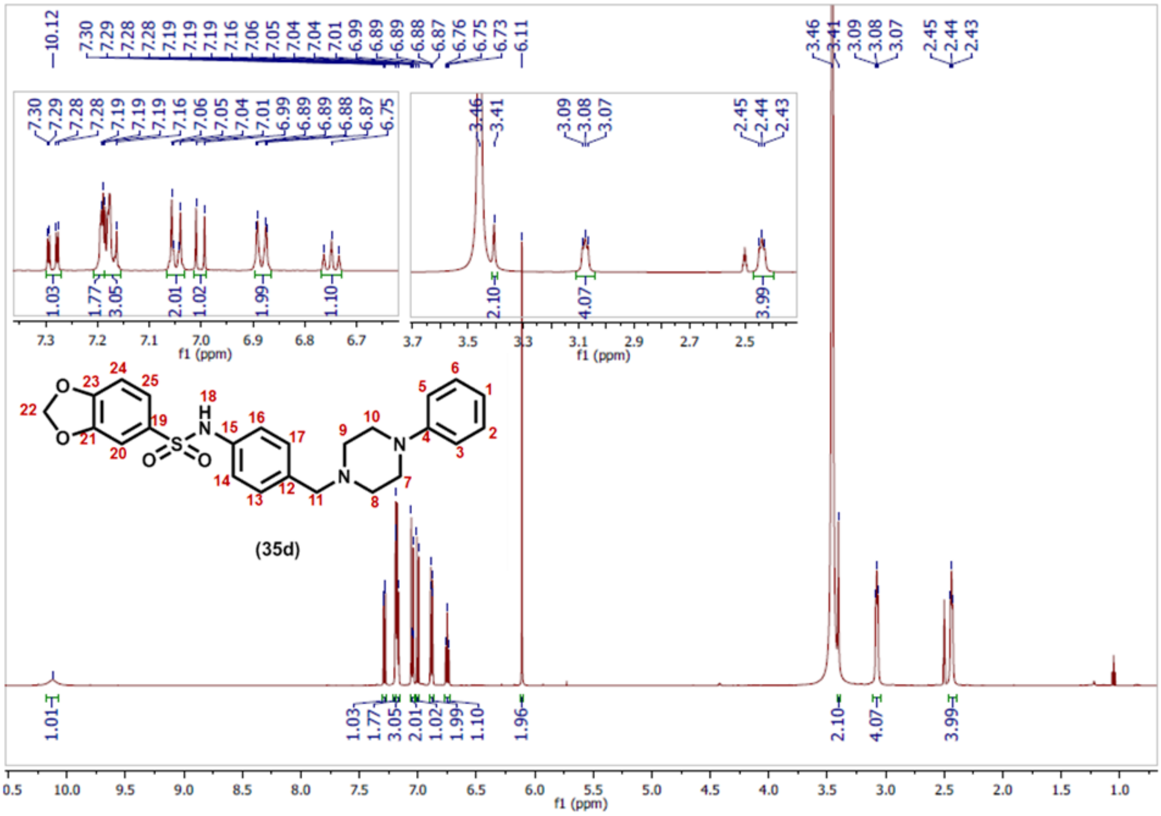

Figure S13: NMR ^1H (500 MHz, 25˚C, DMSO-d_6) of **5d**.

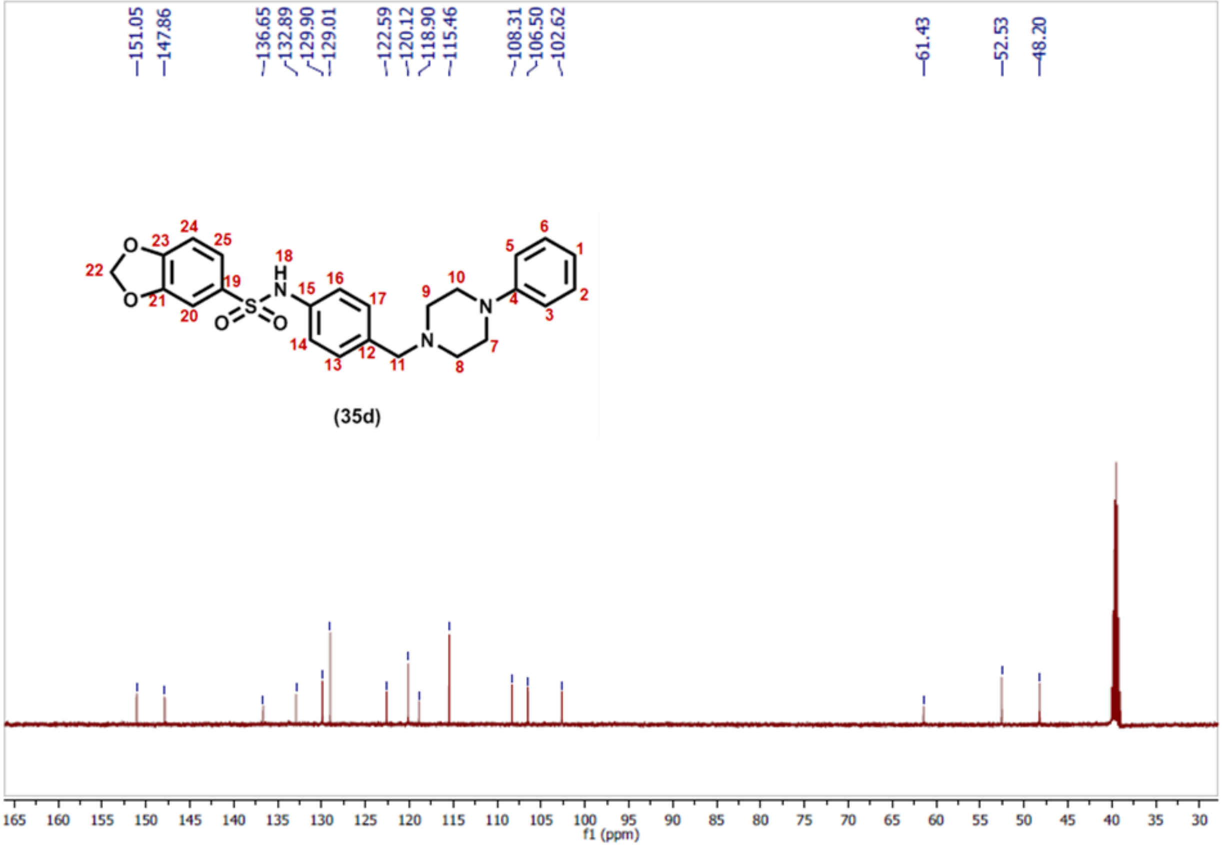

Figure S14: NMR ^{13}C (500 MHz, 25˚C, DMSO-d_6) of **5d**.

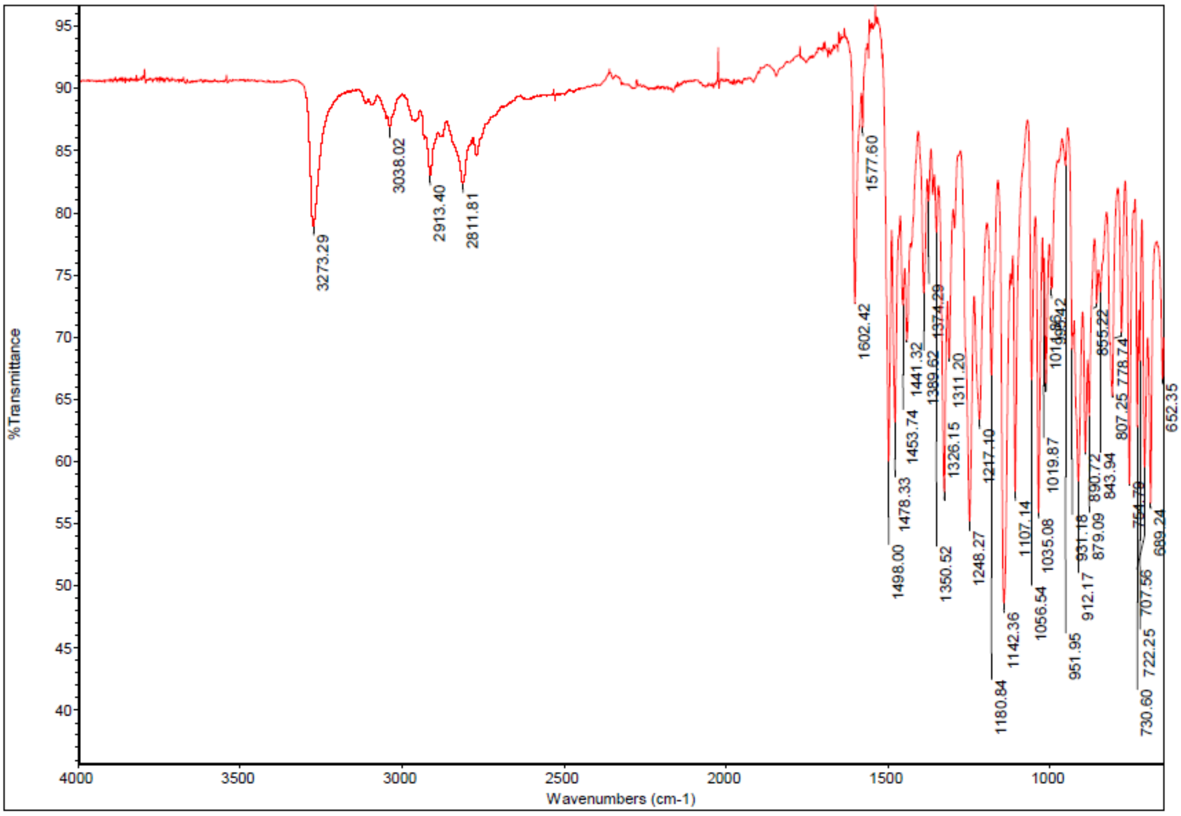

Figure S15: Infrared (ATR) of **5d**.

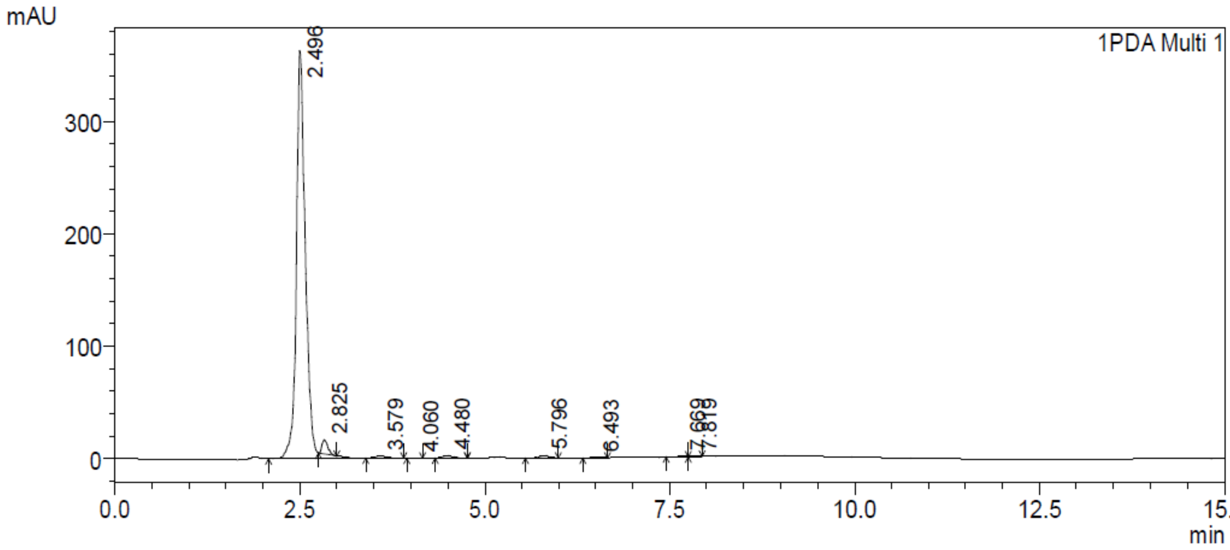

Figure S16: Reverse phase chromatogram in ACN:H2O (60:40) at 287 nm of **5d**.

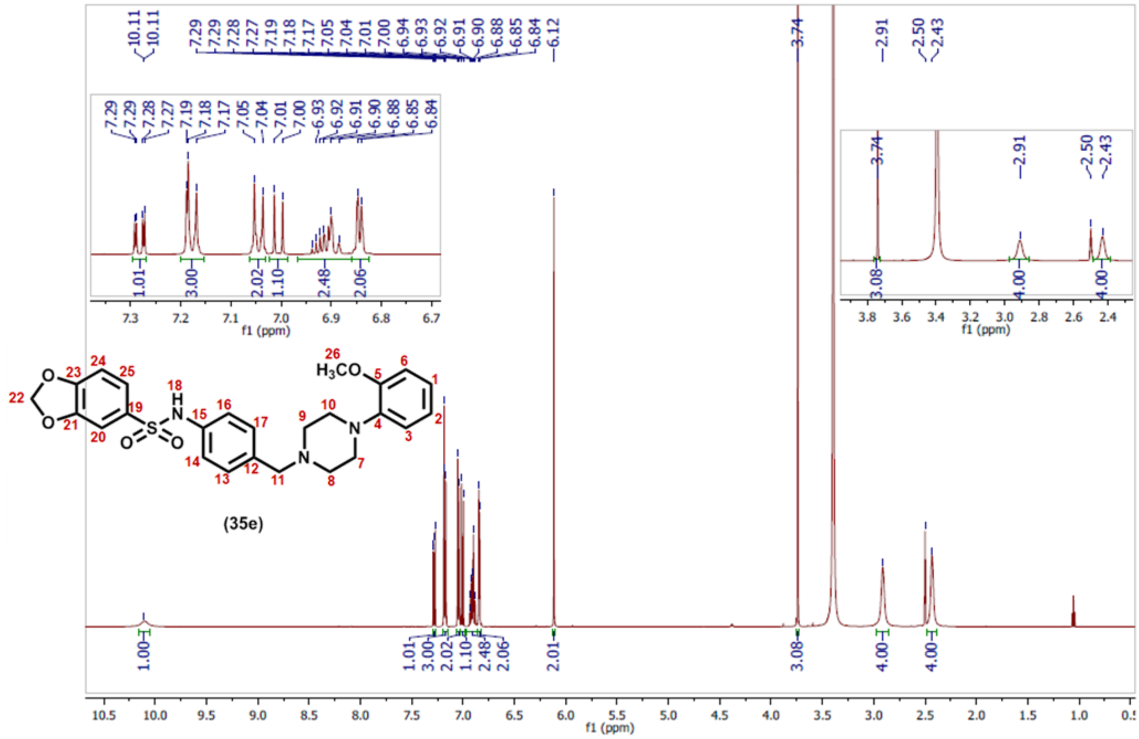

Figure S17: NMR ^{1}H (500 MHz, 25˚C, DMSO-d_6) of **5e**.

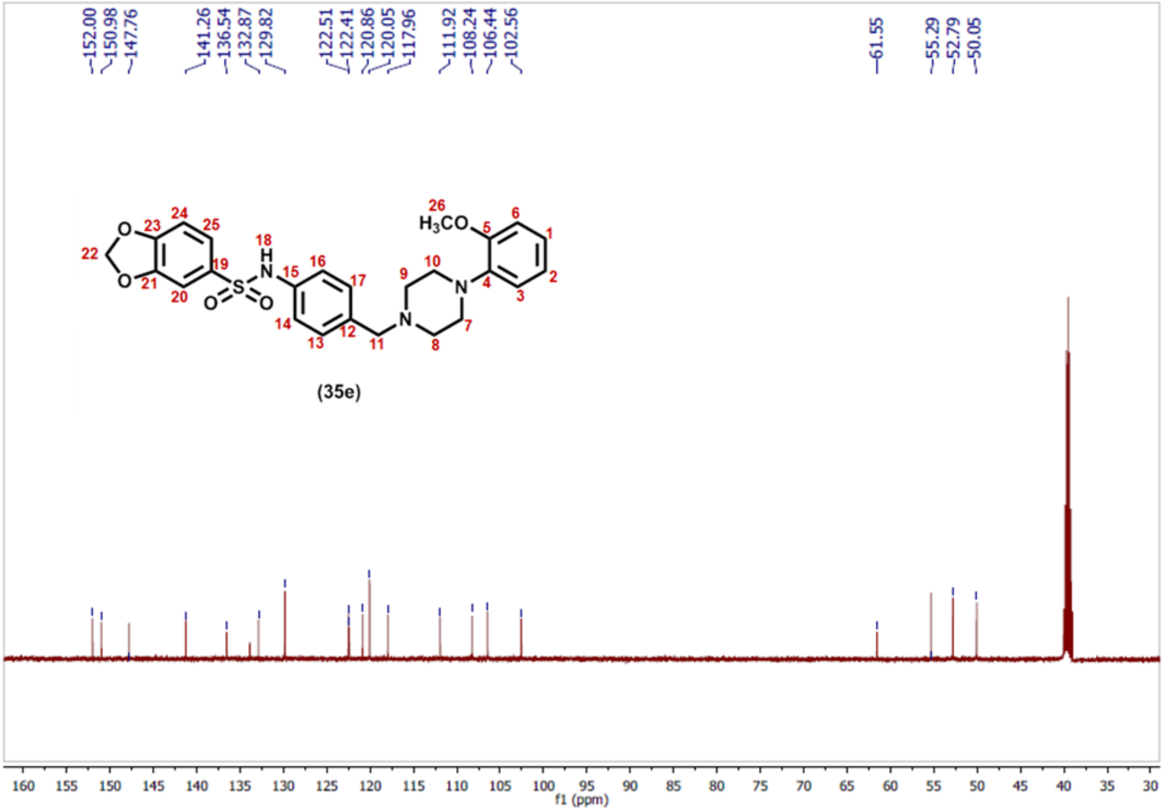

Figure S18: NMR ^{13}C (500 MHz, 25˚C, DMSO-d_6) of **5e**.

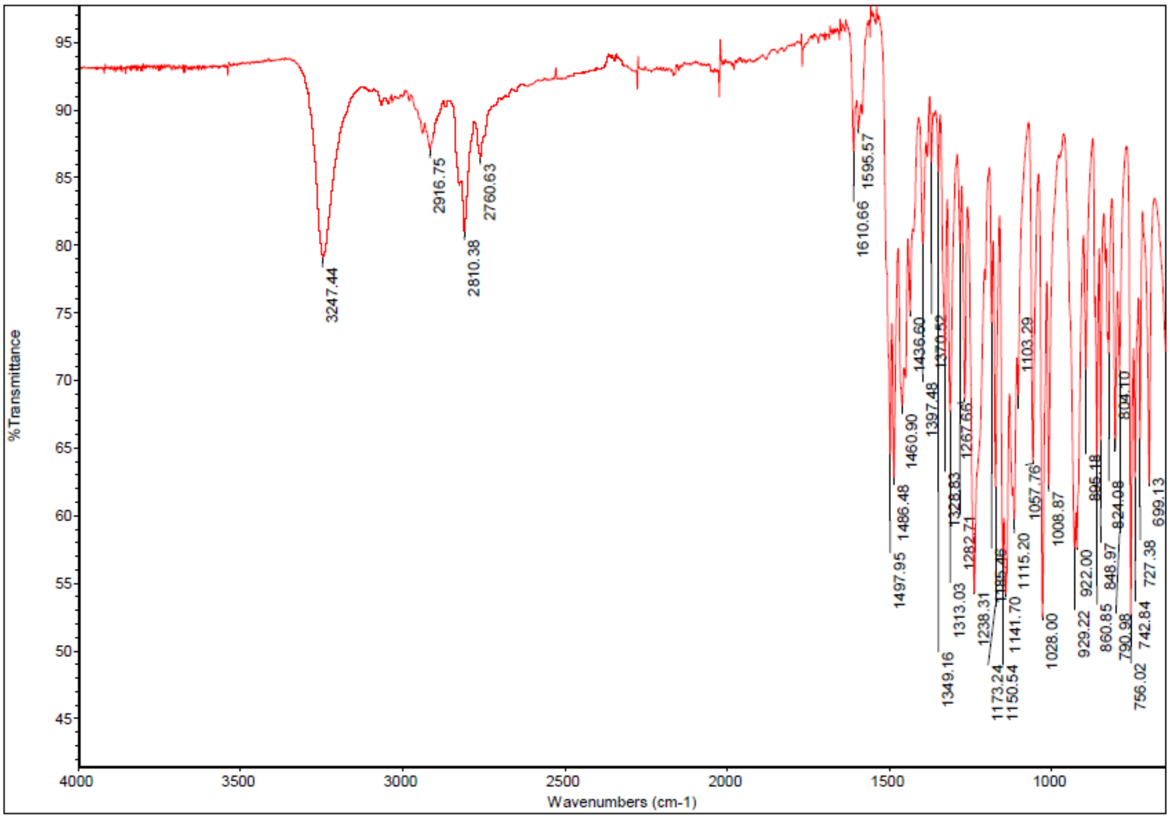

Figure S19: Infrared (ATR) of **5e**.

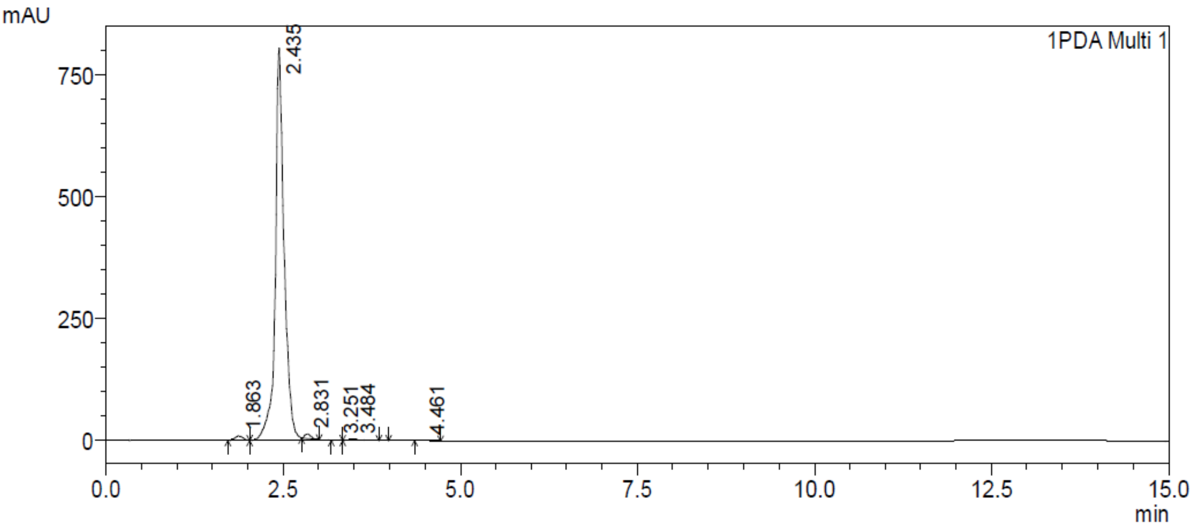

Figure S20: Reverse phase chromatogram in ACN:H2O (60:40) at 284 nm of **5d**.

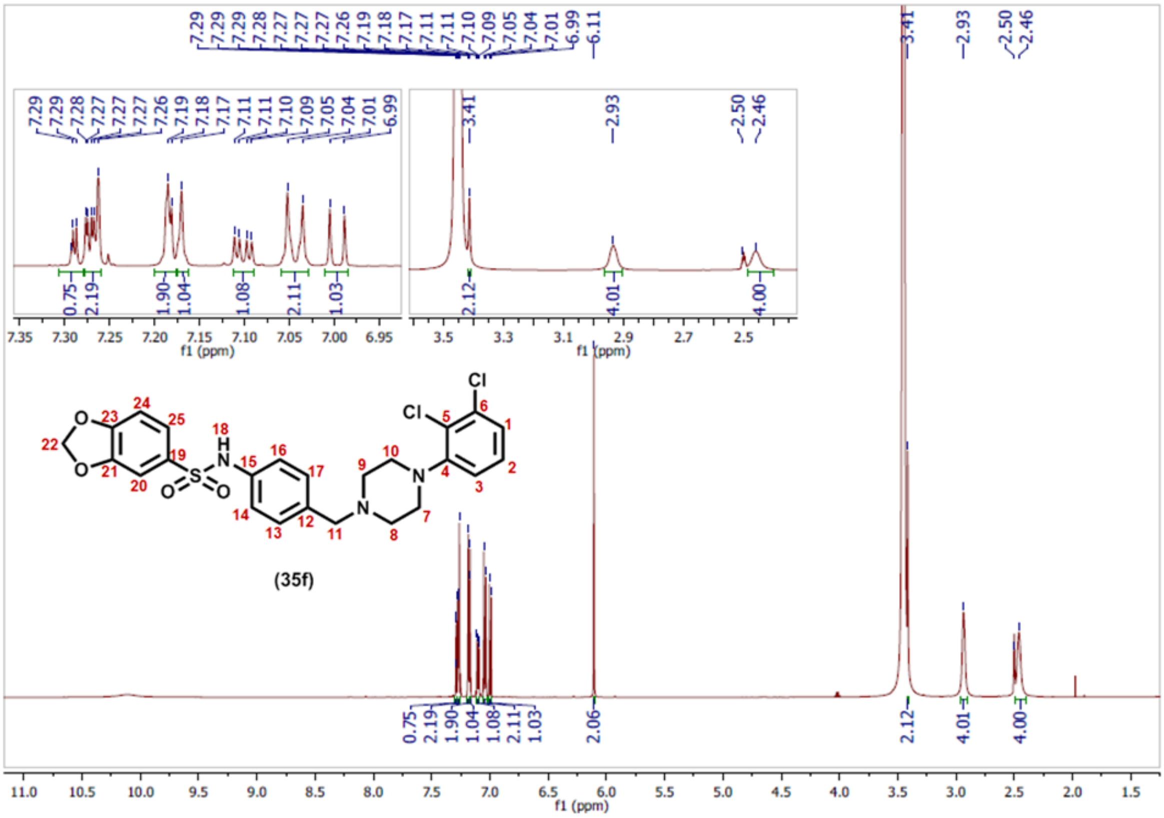

Figure S21: NMR ^{1}H (500 MHz, 25˚C, DMSO-d_6) of **5f**.

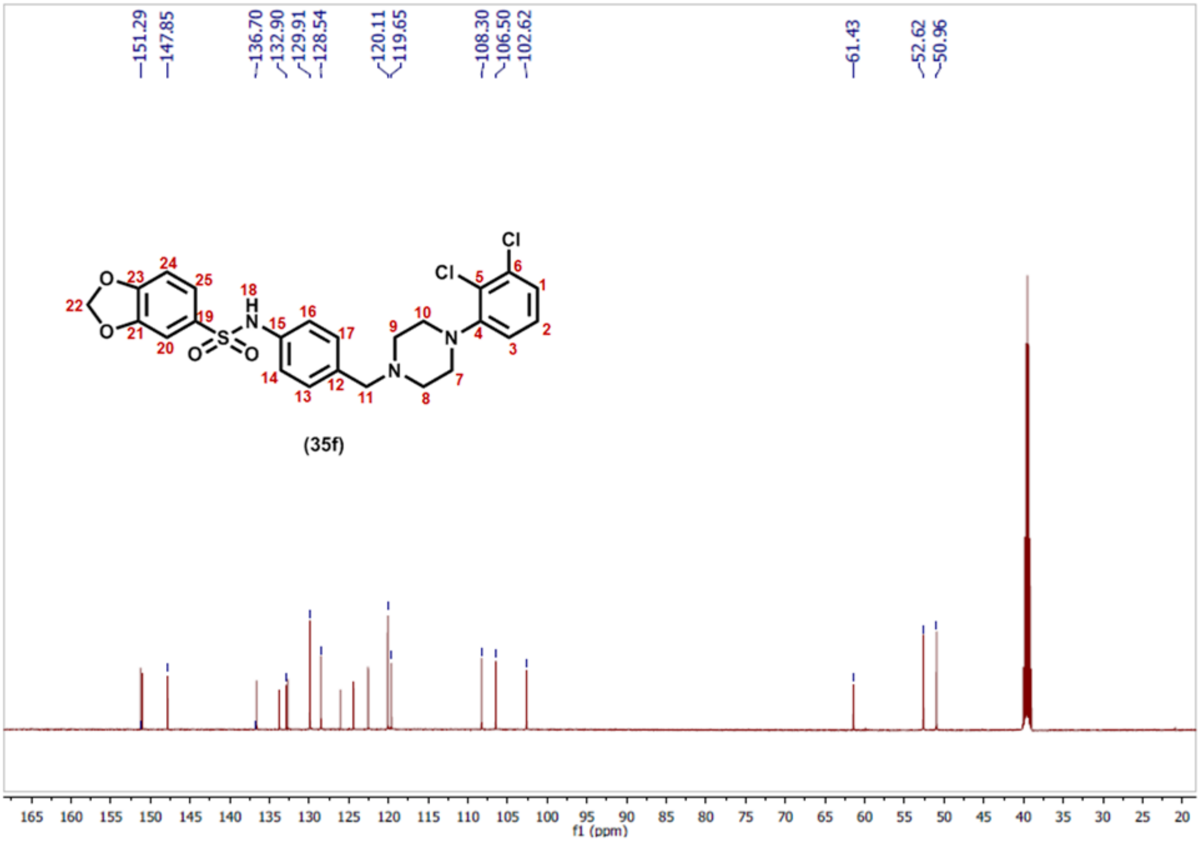

Figure S22: NMR ^{13}C (500 MHz, 25˚C, DMSO-d_6) of **5f**.

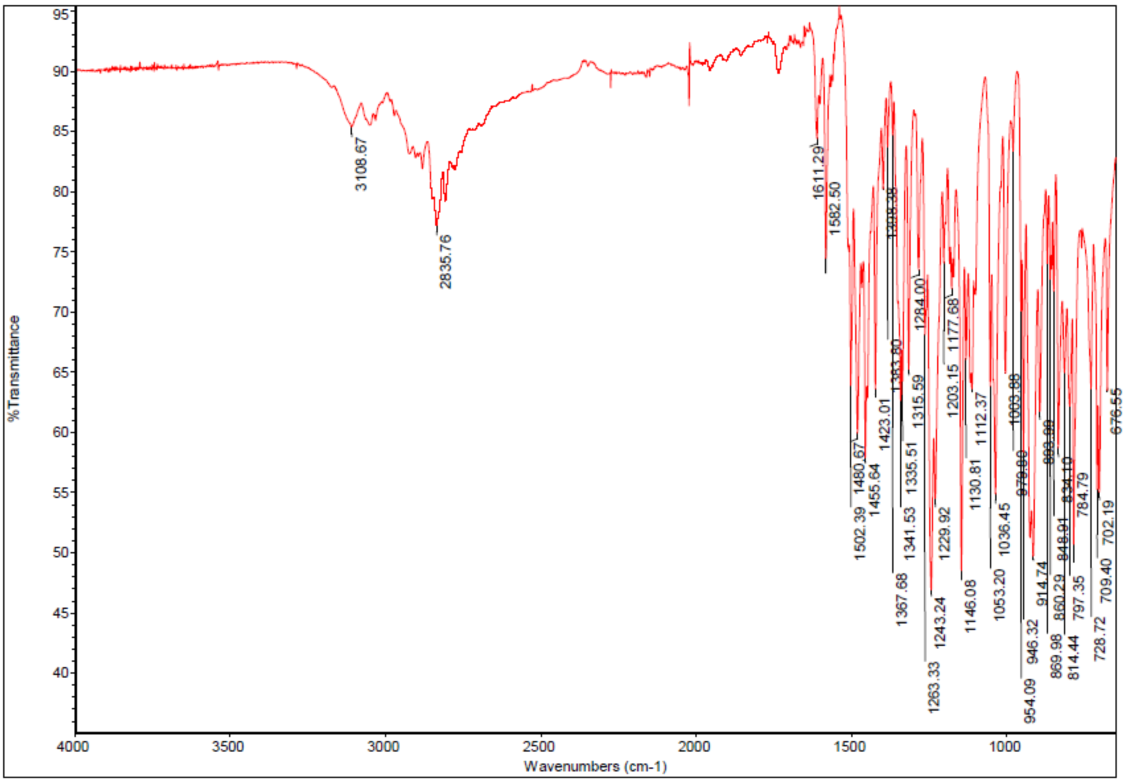

Figure S23: Infrared (ATR) of **5f**.

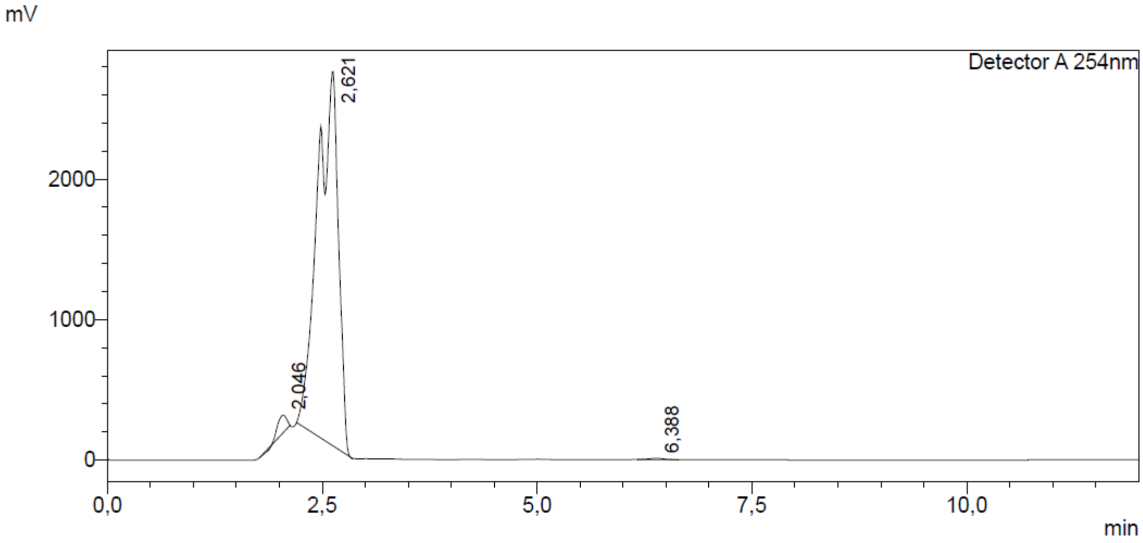

Figure S24: Reverse phase chromatogram in MetOH:H2O (60:40) at 254 nm of **5f**.

2. Molecular Modeling

The four scoring functions present in the GOLD Program (ChemScore [22, 23], GoldScore [24, 25], ChemPLP [26] and ASP [27]) were evaluated by re-docking the co-crystallized ligand to identify the most appropriate function for molecular docking studies of aripiprazole, cariprazine and new derivatives.

The results obtained for mean square deviation (Root Mean Square Deviation-RMSD), can be observed in Table S1.

Table S1: RMSD values for each of the GOLD program function.

Function	Score
ChemPLP	0.387
ChemScore	0.527
GoldScore	1.599
ASP	0.508

In all cases, they presented values lower than the resolution of the crystals. Even so, the function that presented the lowest RMSD value and that, therefore, showed the closest result to the experimental one was ChemPLP.

In Figure S25 it is possible to visually analyze the overlap between the crystallographic structure complex (6CM4) and the complex resulting from the redocking of risperidone using the validated methodology.

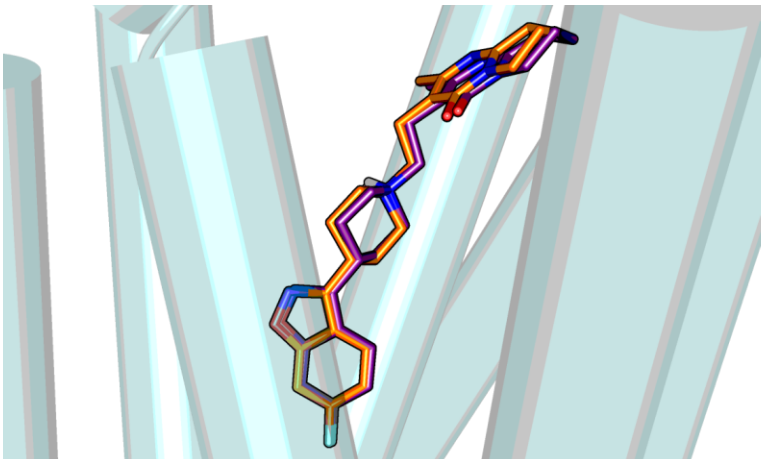

Figure S25: Overlap of risperidone structure of the crystallographic structure (PDB 6CM4) in orange, and the result obtained after redocking by the ChemPLP function in purple.

Aripiprazole, cariprazine and the new derivatives were docked using the ChemPLP function for D_2 and D_3 receptors. The calculations were performed in triplicate, selecting the complexes with the highest score. The scores for each compound can be seen in Table S2.

Table S2: Values of the scores of the results obtained through the docking calculation from the GOLD program, using the ChemPLP function.

Composto	Receptor	
	D_3	D_2
Aripiprazole		
Cariprazine		
5a	73.3	90.8
5b	73.6	87.9
5c	73.6	79.8
5d	78	68.6
5e	84.6	89.1
5f	74.7	67.6

The interpretation of the results obtained by the docking studies was discussed in the main document, using aripiprazole, cariprazine and compound 5a, as an example of the new series obtained. In figures S26 (A-D) and S27 (A-F) we can see the interaction profile identified for the other compounds (5b-f).

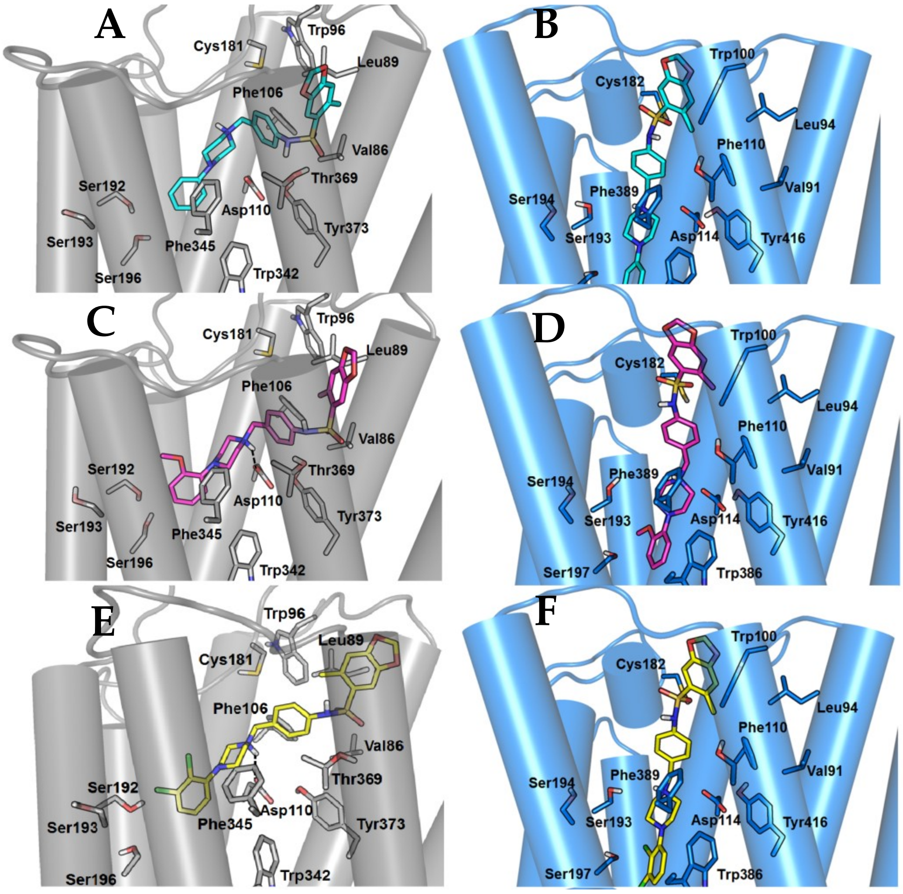

Figure S26: Interaction profile of the proposed compounds on D_3 (grey) and D_2 (blue) receptors. A and B: 5a (light blue); C and D: 5b (pink); E and F: 5c (yellow).

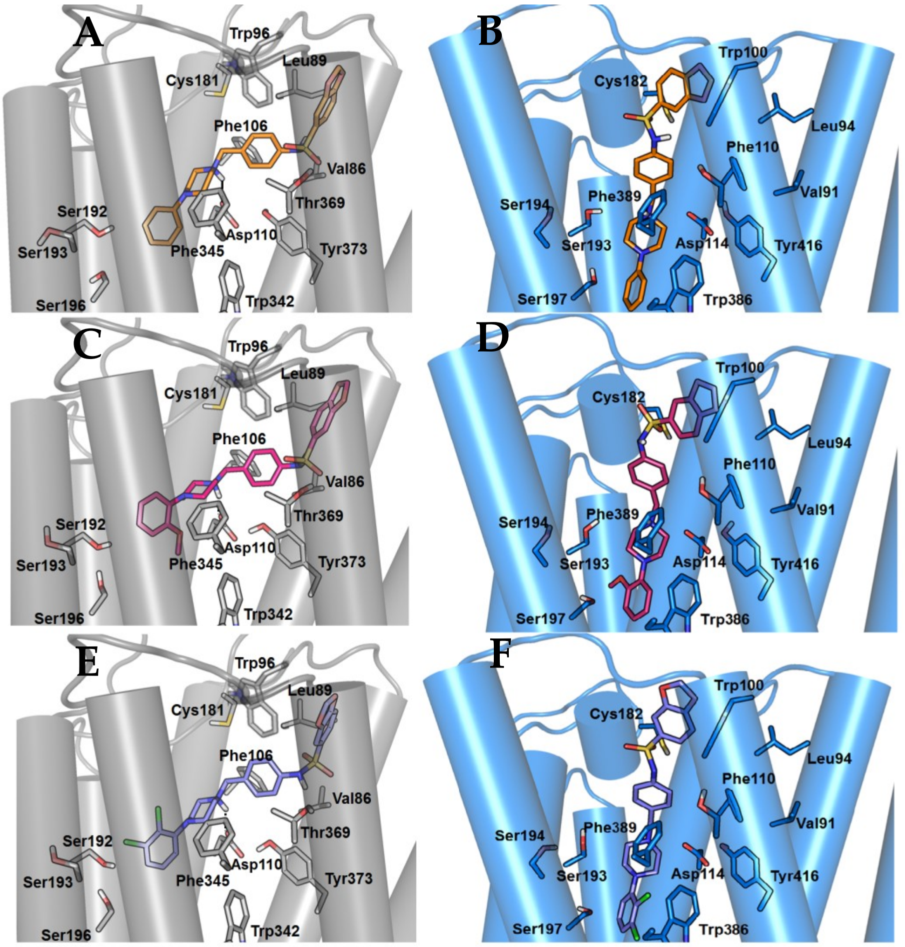

Figure S27: Interaction profile of the proposed compounds on D_3 (grey) and D_2 (blue) receptors. A and B: 5d (orange); C and D: 5e (pink); E and F: 5f (purple).

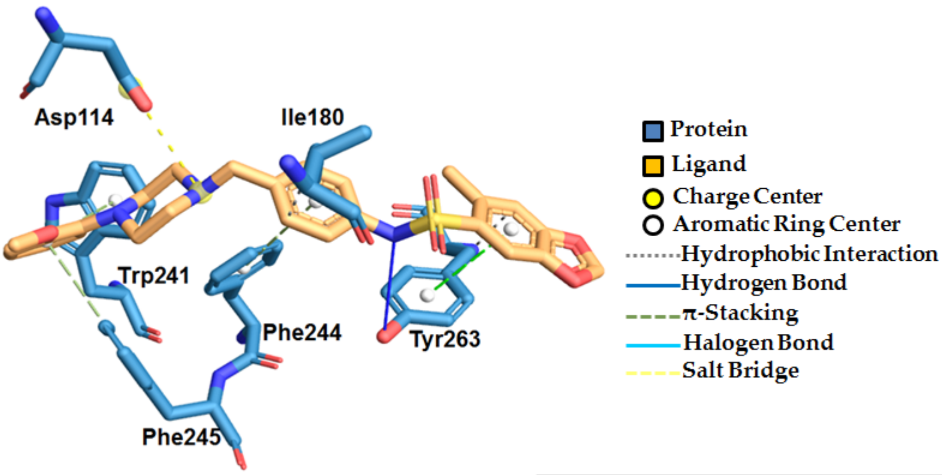

OBS – D2		
Residue	Interaction type	Distance (Å)
Asp114	Salt bridge	4.22
Trp241	π-stacking	4.67
Phe245	π-stacking	5.07

SBP – D2		
Residue	Interaction type	Distance (Å)
Ile180	Hydrogen bond	3.84
Phe244	π-stacking	5.12
Tyr263	Hydrophobic	3.57
Tyr263	Hydrogen bond	2.82 (H-A)/ 3.74 (D-A)

Figure S28: PLIP analysis for the compound 5b, at D_2 receptors.

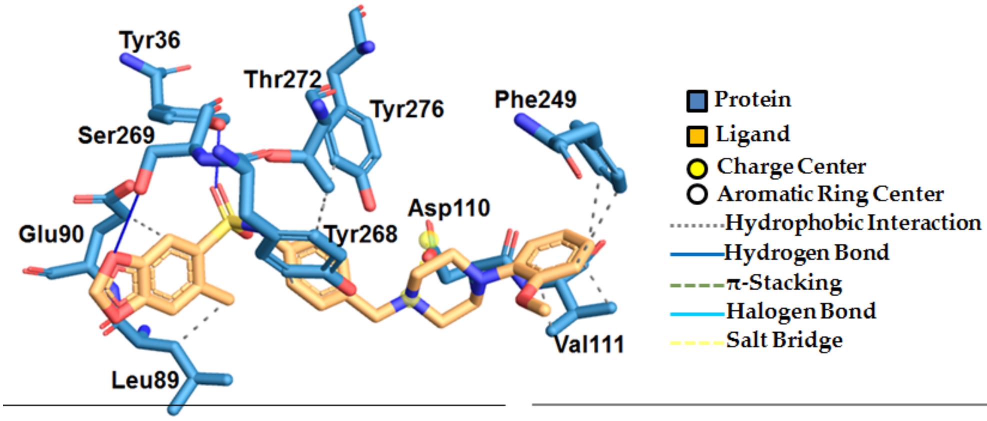

OBS – D3		
Residue	Interaction type	Distance (Å)
Asp110	Salt bridge	3.63
Val111	Hydrophobic	3.87
Phe249	Hydrophobic	3.45

SBP – D3		
Residue	Interaction type	Distance (Å)
Tyr36	Hydrogen bond	1.65 (H-A)/ 2.63 (D-A)
Leu89	Hydrophobic	3.43
Glu90	Hydrophobic	3.87
Tyr268	Hydrophobic	3.64
Ser269	Hydrogen bond	2.66 (H-A)/ 3.41 (D-A)
Thr272	Hydrophobic	3.69
Tyr276	Hydrophobic	3.91

Figure S29: PLIP analysis for the compound 5b, at D_3 receptors.

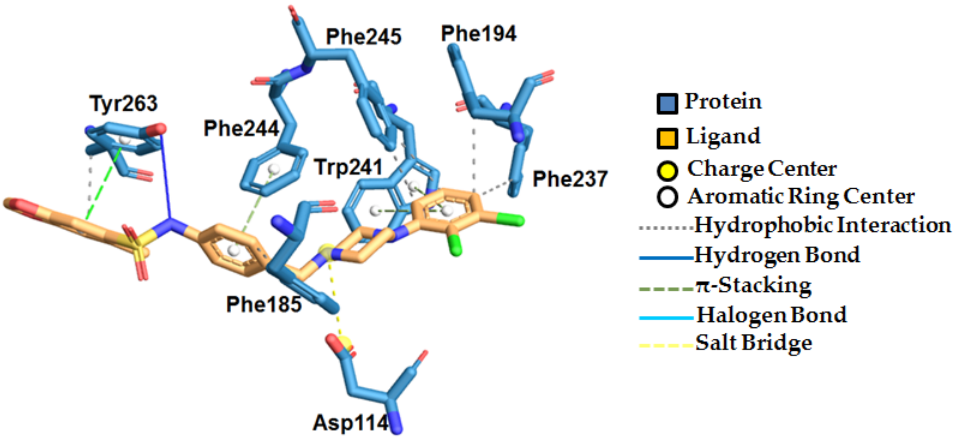

OBS – D2		
Residue	Interaction type	Distance (Å)
Asp114	Salt bridge	4.60
Phe185	Hydrophobic	3.50
Phe194	Hydrophobic	3.85
Phe237	Hydrophobic	3.94
Trp241	π-stacking	4.69
Phe245	Hydrophobic	3.25

SBP – D2		
Residue	Interaction type	Distance (Å)
Phe244	π-stacking	4.87
Tyr263	π-stacking	4.19

Figure S30: PLIP analysis for the compound 5c, at D_2 receptors.

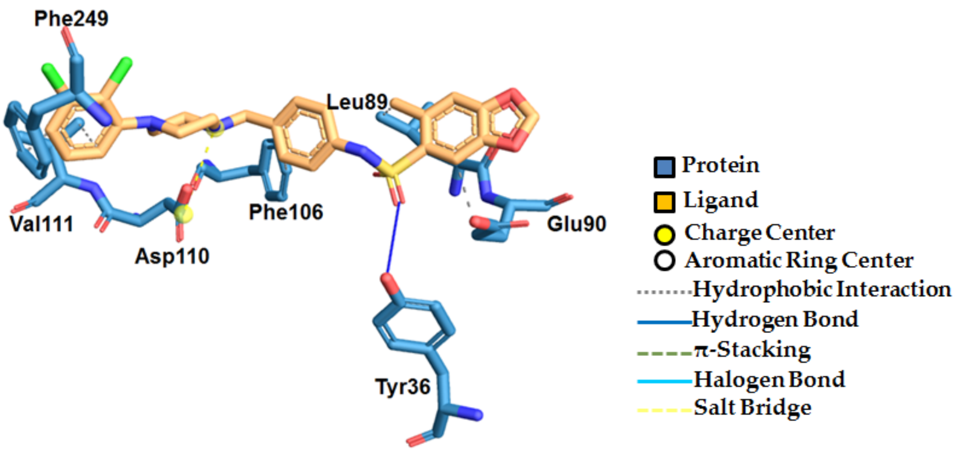

OBS – D3		
Residue	Interaction type	Distance (Å)
Asp110	Salt bridge	3.93
Val111	Hydrophobic	3.71
Phe249	Hydrophobic	3.44

SBP – D3		
Residue	Interaction type	Distance (Å)
Tyr36	Hydrogen bond	3.72 (H-A)/ 4.08 (D-A)
Leu89	Hydrophobic	3.28
Glu90	Hydrophobic	3.38
Phe106	Hydrophobic	3.69

Figure S31: PLIP analysis for the compound 5c, at D_3 receptors.

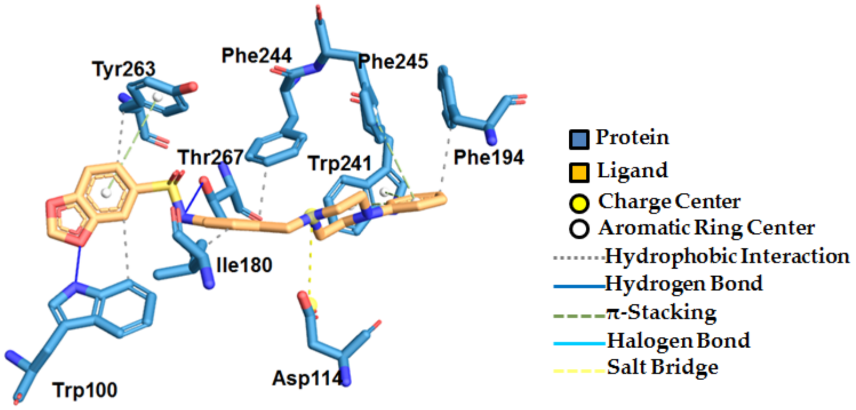

OBS – D2		
Residue	Interaction type	Distance (Å)
Asp114	Salt bridge	4.63
Phe194	Hydrophobic	3.70
Trp241	π-stacking	4.82
Phe245	π-stacking	4.93

SBP – D2		
Residue	Interaction type	Distance (Å)
Trp100	Hydrophobic	3.85
Trp100	Hydrogen bond	1.75 (H-A)/ 2.73 (D-A)
Ile180	Hydrophobic	3.06
Phe244	Hydrophobic	3.31
Tyr263	Hydrophobic	3.85
Tyr263	π-stacking	5.35
Thr267	Hydrophobic	3.35
Thr267	Hydrogen bond	2.84 (H-A)/ 3.80 (D-A)

Figure S32: PLIP analysis for the compound 5d, at D_2 receptors.

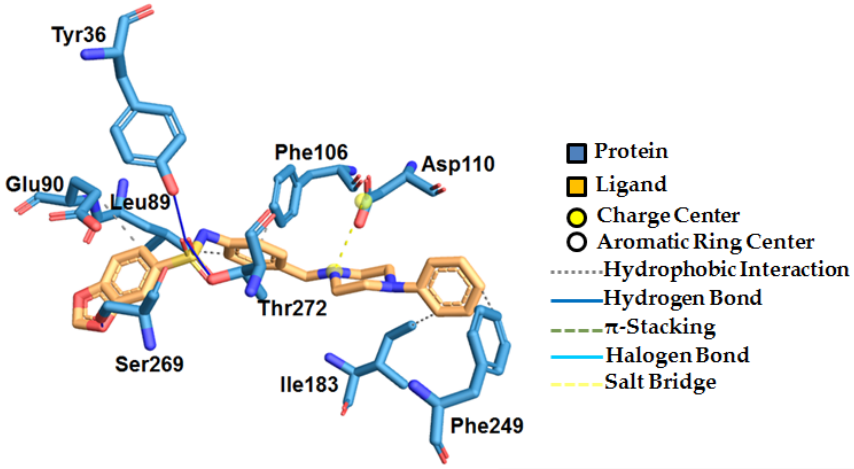

OBS – D3		
Residue	Interaction type	Distance (Å)
Asp110	Salt bridge	3.86
Ile183	Hydrophobic	3.80
Phe249	Hydrophobic	3.49

SBP – D3		
Residue	Interaction type	Distance (Å)
Tyr36	Hydrogen bond	2.09 (H-A)/ 3.03 (D-A)
Leu89	Hydrophobic	3.94
Glu90	Hydrophobic	3.48
Phe106	Hydrophobic	3.57
Thr272	Hydrogen bond	2.09 (H-A)/ 3.05 (D-A)
Ser269	Hydrogen bond	2.75 (H-A)/ 3.10 (D-A)

Figure S33: PLIP analysis for the compound 5d, at D_3 receptors.

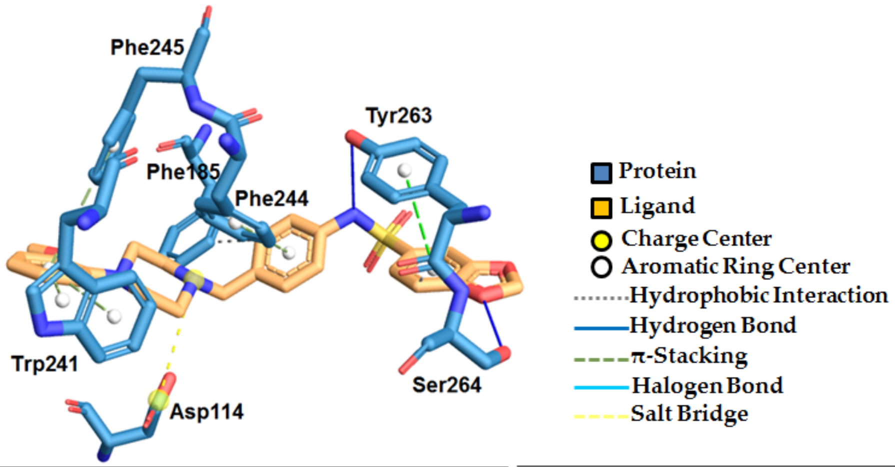

OBS – D2		
Residue	Interaction type	Distance (Å)
Asp114	Salt bridge	4.56
Phe185	Hydrophobic	3.56
Trp241	π-stacking	4.66/ 5.01
Phe244	π-stacking	5.11
Phe245	π-stacking	5.03

SBP – D2		
Residue	Interaction type	Distance (Å)
Tyr263	Hydrophobic	3.92
Tyr263	Hydrogen bond	2.73 (H-A)/ 3.44 (D-A)
Tyr263	π-stacking	4.17
Ser264	Hydrogen bond	1.92 (H-A)/ 2.81 (D-A)

Figure S34: PLIP analysis for the compound 5e, at D_2 receptors.

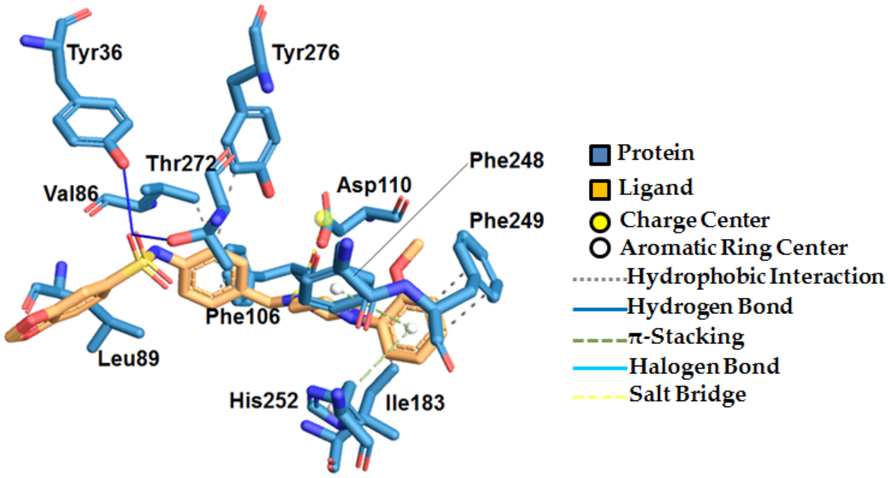

OBS – D3		
Residue	Interaction type	Distance (Å)
Asp110	Salt bridge	3.66
Ile183	Hydrophobic	3.55
Phe248	π-stacking	4.80
Phe249	Hydrophobic	3.60
His252	π-stacking	4.91

SBP – D3		
Residue	Interaction type	Distance (Å)
Tyr36	Hydrogen bond	2.08 (H-A)/ 3.02 (D-A)
Val86	Hydrophobic	3.57
Leu89	Hydrophobic	3.64
Phe106	Hydrophobic	3.41
Thr272	Hydrogen bond	2.11 (H-A)/ 3.07 (D-A)
Tyr276	Hydrogen bond	3.77

Figure S35: PLIP analysis for the compound 5e, at D_3 receptors.

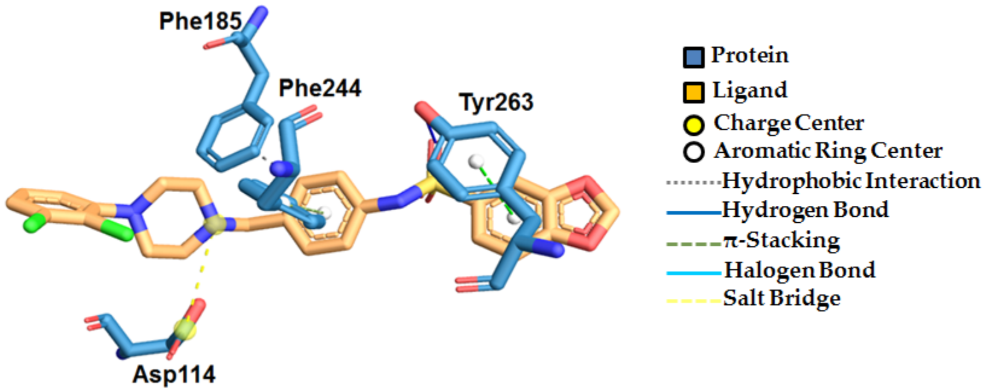

OBS – D2		
Residue	Interaction type	Distance (Å)
Asp114	Salt bridge	4.11
Phe185	Hydrophobic	3.56
Phe244	π-stacking	4.94

SBP – D2		
Residue	Interaction type	Distance (Å)
Tyr263	Hydrogen bond	2.24 (H-A)/ 3.16 (D-A)
Tyr263	π-stacking	3.90

Figure S36: PLIP analysis for the compound 5f, at D_2 receptors.

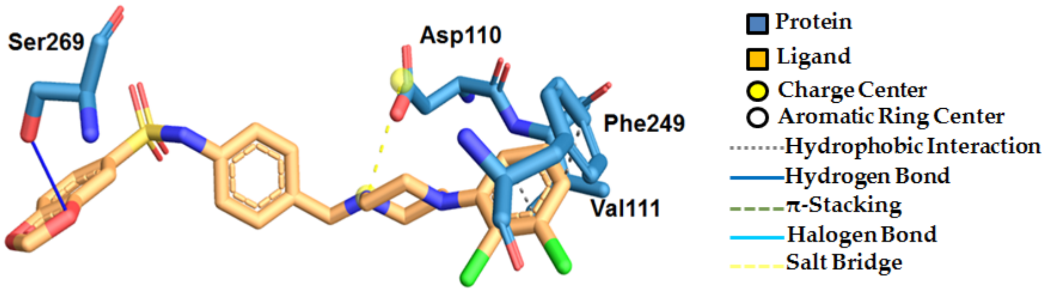

OBS – D3		
Residue	Interaction type	Distance (Å)
Asp110	Salt bridge	3.90
Val111	Hydrophobic	3.78
Phe249	Hydrophobic	3.36

SBP – D3		
Residue	Interaction type	Distance (Å)
Ser269	Hydrogen bond	2.09 (H-A)/ 2.86 (D-A)

Figure S37: PLIP analysis for the compound 5f, at D_3 receptors.

3. Binding and GTP-shift

Effect of compounds 5a-f on [^{3}H]-YM-09151-2 binding to D_2 receptor (D_2R) present in rat striatum membrane preparation (Figure S38).

In the main document, we present the competition curves for dopamine (full agonist) for validation purposes. We also show the competition curve for compound 5a as a way of discussing functional binding assays. The competition curves for the other synthesized compounds are shown in Figure S39 (A-F).

It was not possible to obtain the competition curve for compound 5f, because the experiment for this compound was carried out in duplicate.

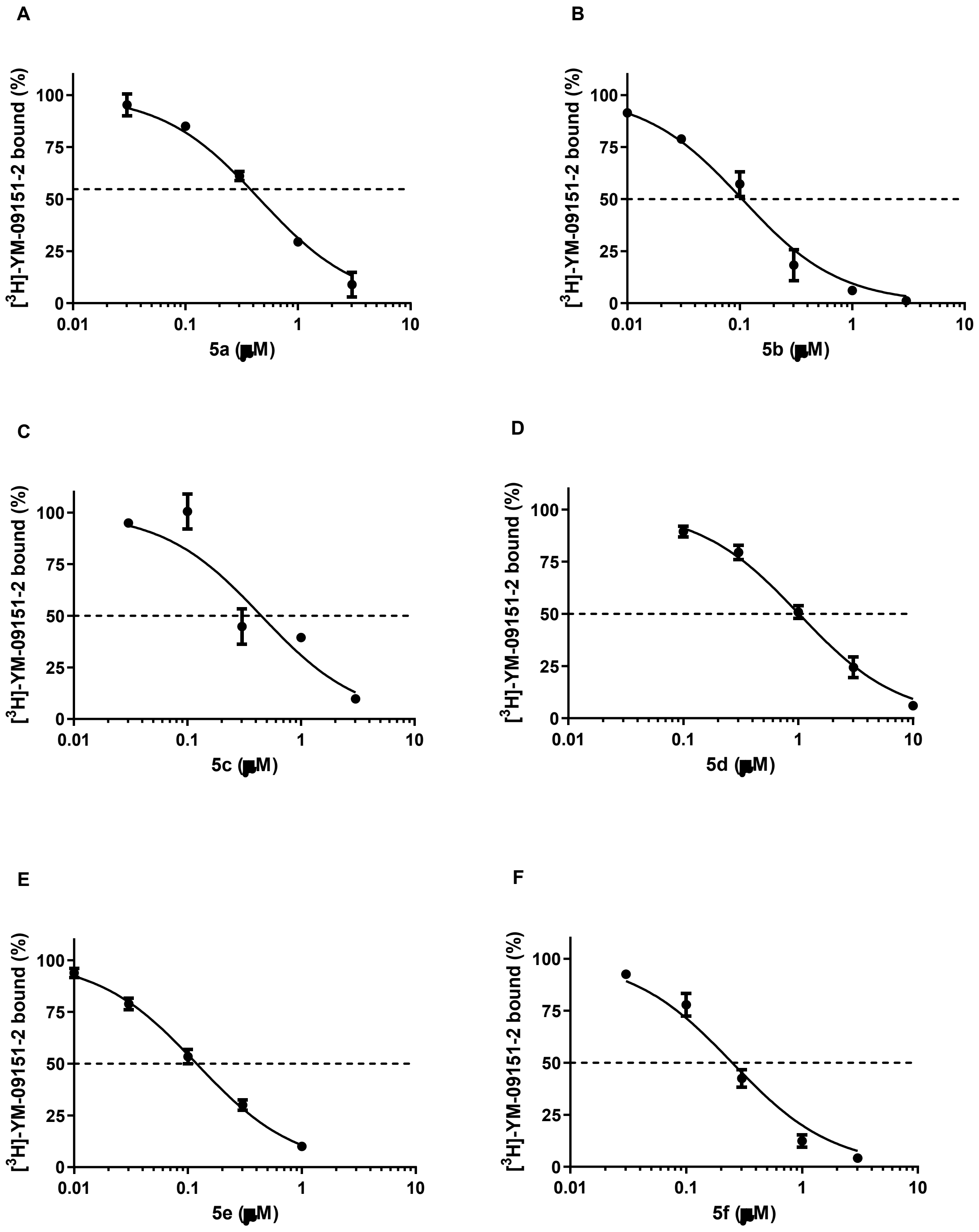

Figure S38: Estimation of the affinity of of 5a (A), 5b (B), 5c (C), 5d (D), 5e (E) and 5f (F) on [^{3}H]-YM-09151-2 binding to rat striatal D2 receptor (D2R). Data are means (±S.E.) from two or three independent experiments, each performed in triplicate. The data were fitted assuming a single population of binding sites and curves were drawn using the parameters fitted by nonlinear regression (see details in the Materials and Methods).

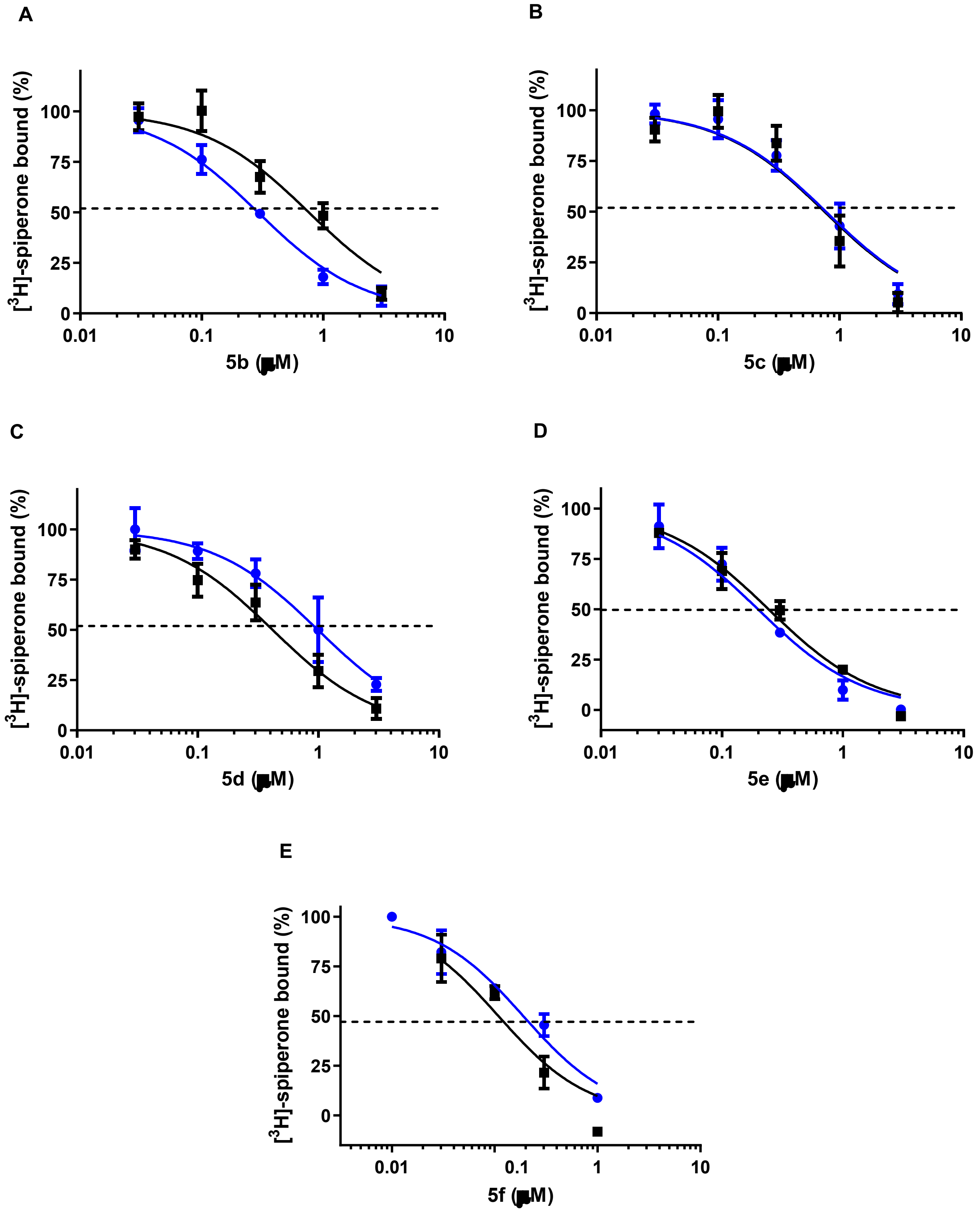

Figure S39: Estimation of the intrinsic efficacy of 5b (A), 5c (B), 5d (C), 5e (D), and 5f (E) at the human D_3 in membrane preparations of recombinant Chem-1 cells. Competition curves were performed using the antagonist radioligand (0.5 nM [^{3}H]-spiperone) in the presence of 5 mM $MgCl_2$ and 1.5 mM $CaCl_2$ (black) or 154 mM NaCl and 1 mM GTP (blue). Each curve represents the averaged curve (±S.E.) from two or three independent paired experiments (in triplicate).
